# CAGE-Seq Reveals that HIV-1 Latent Infection Does Not Trigger Unique Cellular Responses in a Jurkat T Cell Model

**DOI:** 10.1128/JVI.02394-20

**Published:** 2021-03-25

**Authors:** Hiroyuki Matsui, Kotaro Shirakawa, Yoshinobu Konishi, Shigeki Hirabayashi, Anamaria Daniela Sarca, Hirofumi Fukuda, Ryosuke Nomura, Emani Stanford, Yoshihito Horisawa, Yasuhiro Kazuma, Tadahiko Matsumoto, Hiroyuki Yamazaki, Yasuhiro Murakawa, Emilie Battivelli, Eric Verdin, Yoshio Koyanagi, Akifumi Takaori-Kondo

**Affiliations:** a Department of Hematology and Oncology, Graduate School of Medicine, Kyoto University, Kyoto, Japan; b Department of Hematology, Kansai Electric Power Medical Research Institute, Osaka, Japan; c RIKEN Center for Integrative Medical Sciences, Yokohama, Japan; d Institute for the Advanced Study of Human Biology, Kyoto University, Kyoto, Japan; e Department of Medical Systems Genomics, Graduate School of Medicine, Kyoto University, Kyoto, Japan; f The FIRC Institute of Molecular Oncology (IFOM), Milan, Italy; g Buck Institute for Research on Aging, Novato, California, USA; h Laboratory of Systems Virology, Institute for Frontier Life and Medical Sciences, Kyoto University, Kyoto, Japan; Ulm University Medical Center

**Keywords:** CAGE-seq, HIV-1, gene expression, latency

## Abstract

The cure for HIV-1 is currently stalled by our inability to specifically identify and target latently infected cells. HIV-1 viral RNA/DNA or viral proteins are recognized by cellular mechanisms and induce interferon responses in virus-producing cells, but changes in latently infected cells remain unknown. HIV_GKO_ contains a green fluorescent protein (GFP) reporter under the HIV-1 promoter and a monomeric Kusabira orange 2 (mKO2) reporter under the internal elongation factor alpha (EF1α) promoter. This viral construct enables direct identification of both productively and latently HIV-1-infected cells. In this study, we aim to identify specific cellular transcriptional responses triggered by HIV-1 entry and integration using cap analysis of gene expression (CAGE). We deep sequenced CAGE tags in non-infected and latently and productively infected cells and compared their differentially expressed transcription start site (TSS) profiles. Virus-producing cells had differentially expressed TSSs related to T-cell activation and apoptosis compared to those of non-infected cells or latently infected cells. Surprisingly, latently infected cells had only 33 differentially expressed TSSs compared to those of non-infected cells. Among these, SPP1 and APOE were downregulated in latently infected cells. SPP1 or APOE knockdown in Jurkat T cells increased susceptibility to HIV_GKO_ infection, suggesting that they have antiviral properties. Components of the phosphatidylinositol 3-kinase (PI3K)/mammalian target of rapamycin (mTOR) pathway, MLST8, 4EBP, and RPS6, were significant TSSs in productively infected cells, and S6 kinase (S6K) phosphorylation was increased compared to that in latently infected cells, suggesting that mTOR pathway activity plays a role in establishing the latent reservoir. These findings indicate that HIV-1 entry and integration do not trigger unique transcriptional responses when infection becomes latent.

**IMPORTANCE** Latent HIV-1 infection is established as early as the first viral exposure and remains the most important barrier in obtaining the cure for HIV-1 infection. Here, we used cap analysis of gene expression (CAGE) to compare the transcriptional landscape of latently infected cells with that of non-infected or productively infected cells. We found that latently infected cells and non-infected cells show quite similar transcriptional profiles. Our data suggest that T cells cannot recognize incoming viral components or the integrated HIV-1 genome when infection remains latent. These findings should guide future research into widening our approaches to identify and target latent HIV-1-infected cells.

## Abstract (precis)

Latent HIV-1 infection is established as early as the first viral exposure and remains the most important barrier in obtaining the cure for HIV-1 infection. Here, we used cap analysis of gene expression (CAGE) to compare the transcriptional landscape of latently infected cells with that of noninfected or productively infected cells.

## INTRODUCTION

HIV-1 infection has changed from a life-threatening disease to a chronic infectious disease with the introduction of antiretroviral therapy (ART). However, ART cannot eradicate HIV-1, because HIV-1 persists in the human body in a latent state and starts to replicate within weeks after cessation of ART. This latent infection occurs within long-lived cells, including resting CD4^+^ T cells, follicular dendritic cells, and hematopoietic stem cells, and can be established at a very early stage of infection (1). HIV-1 enters the cellular nucleus and integrates into the human genome, targeting mainly the introns of actively transcribed genes close to the nucleopore (2–4). HIV-1 transcription depends on the cellular transcriptional machinery; therefore, the chromatin status of its integration site will greatly affect the viral transcriptional outcome. If integration occurs in heterochromatin or within genes that will later be inactivated, these integration events can lead to latent infection directly after integration (5, 6). Current ART is capable of suppressing ongoing viral transcription and new infections (7–9); therefore, to achieve an HIV cure, it is imperative to elucidate the mechanism behind HIV-1 latent infection and the early events around HIV-1 integration.

Productive HIV-1 infection triggers cellular innate immune responses and affects cellular membrane protein expression. For example, gamma interferon inducible protein 16 (IFI16) and cyclic GMP-AMP synthase (cGAS) recognize HIV reverse transcription (RT) products and activate the adaptor protein STING, which then stimulates TBK1 and interferon regulatory factor 3 (IRF3) to induce the transcription of interferon genes (10, 11). HIV-1 evades this innate immune response by interacting with host proteins such as cyclophilin A (CypA) and polyadenylation specificity factor subunit 6 (CPSF6), which protects RT products from being detected by IFI16 and cGAS (12–14). Although a number of surface markers of productive HIV-1 infection have been identified, including immune checkpoint molecules (15), CD2 (16), CD30 (17), and CD32 (18), there are currently no established surface markers that can distinguish latently infected cells from non-infected cells.

Various HIV latency models using primary CD4^+^ T cells and reporter HIV constructs have been developed for latency research (19). One of these constructs is HIV_GKO_, which contains two fluorescent reporters: codon-switched enhanced green fluorescent protein (csGFP) driven by the HIV-1 long terminal repeat (LTR) promoter and monomeric Kusabira orange 2 (mKO2) driven by an internal elongation factor alpha (EF1α) promoter. HIV_GKO_ enables us to identify latently infected cells directly as mKO2-single-positive cells and distinguish latently infected cells from non-infected and productively infected cells (6, 20).

Single cell RNA-seq results of latently infected cells using primary cell models of HIV-1 latency showed that latently infected cells express some memory T-cell markers (*CCR7*, *SELL*, and *CD27*), and reactivated cells express T-cell activation markers (*IL2RA*, *HLA-DR*, and *CD38*) (21). Most transcriptome studies of HIV-1 latency compared the transcriptome profile of latently infected cells to that of reactivated cells (22, 23). Thus, there are no reports that directly compare the transcriptome profile of latently infected cells to that of non-infected cells.

Cap analysis of gene expression (CAGE) is a method for promoter identification and transcription profiling by deep sequencing the 5′ capped ends of transcripts that can provide robust quantification of 5′ ends of transcripts that exist at low levels (24, 25). In this study, we use CAGE to perform a transcriptome analysis of a cell line model of HIV-1 latency. We found that the transcriptome profile of latently infected cells is quite similar to that of non-infected cells and that latent infection does not induce a specific cellular response. Even though latently infected cells showed a very similar transcriptomic profile to that of non-infected cells, we found that SPP1 and APOE were significantly enriched in non-infected cells compared to that in latently infected cells and that knock down of these genes promoted HIV-1 infection. In contrast, we observed clear differences in the expression levels of three mammalian target of rapamycin (mTOR) signaling pathway genes, *MLST8*, *RPS6*, and *EIF4EBP1*, between latently infected and productively infected cells. We show that RPS6 knockdown increased phosphorylation of S6 kinase (S6K) through negative feedback and resulted in an increase of productive infection. Furthermore, S6K inhibitor treatment increased latent infection, suggesting that S6K activity correlates with HIV-1 transcription and latency formation.

## RESULTS

### Characterization of the latency model using the dual-fluorescence HIV-1 reporter HIV_GKO_.

**FIG 1:**
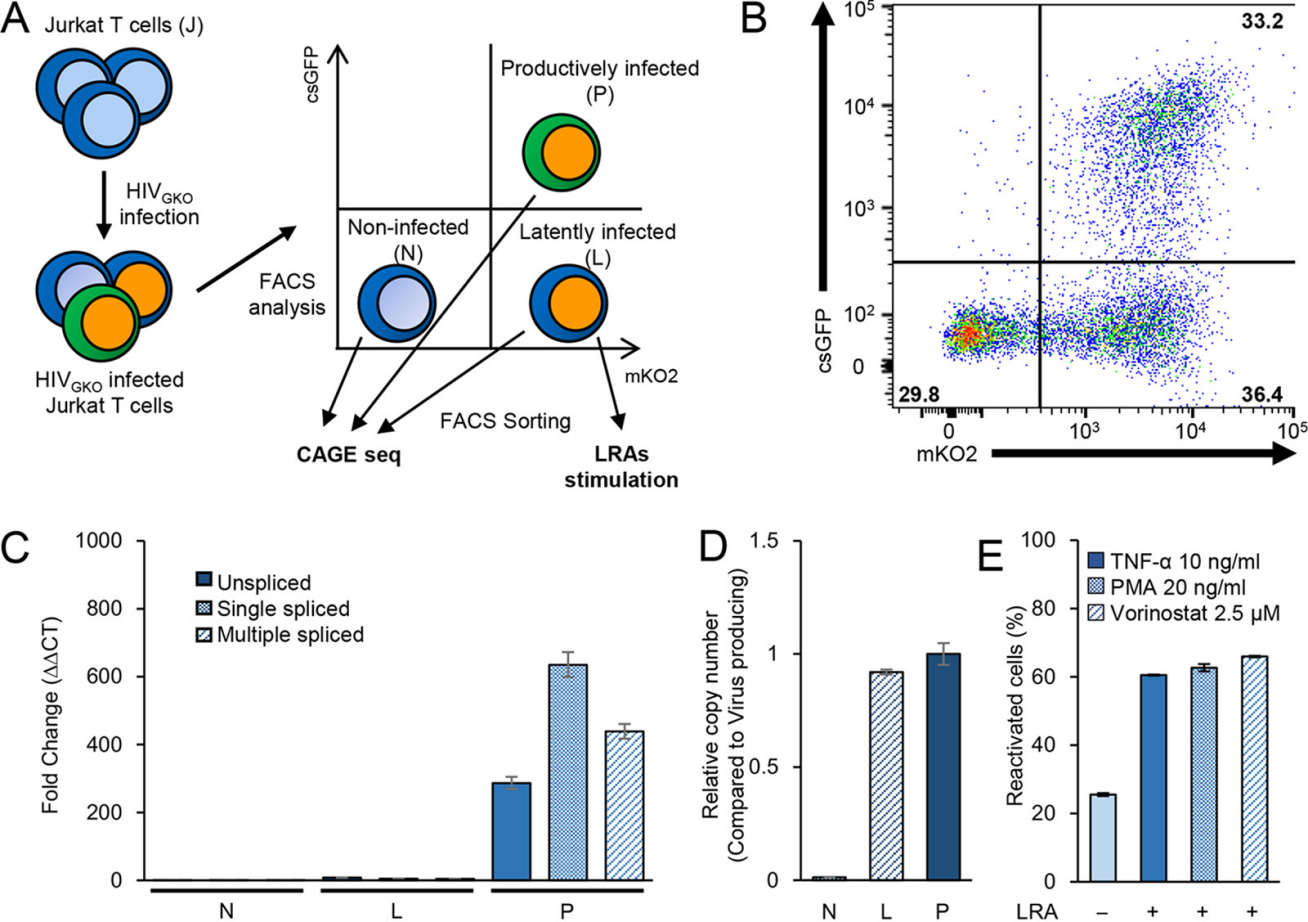
Characterization of the HIV_GKO_ latency model. (A) Schema of the experimental workflow. Briefly, Jurkat T cells were infected with HIV_GKO_ and, 4 days later, were analyzed and sorted with a FACS Aria II. Sorted cells were subjected to CAGE analysis and stimulation with LRAs as indicated. (B) Representative FACS plot and gating at sorting. (C) At 4 days postinfection, we sorted non-infected (N), latently infected (L), and productively infected (P) cells, extracted total RNA, and performed RT-qPCR. Unspliced (US), single-spliced (SS), and multiple-spliced (MS) HIV-1 RNAs were quantified relative to cellular GAPDH and presented as fold change relative to non-infected control (*n* = 3, mean ± standard deviation [SD]). (D) Cells were sorted as shown in panel A. We extracted genomic DNA from each fraction and performed Alu-based quantitative genomic PCR. The copy number of integrated provirus in each fraction is presented relative to that of productively infected cells (*n* = 3, mean ± SD). (E) At 4 days postinfection, latently infected cells were sorted, cultured overnight, and stimulated with different LRAs for 24 h before performing flow cytometry. Percentage of reactivated double-positive (mKO2^+^ GFP^+^) cells is shown (*n* = 2, mean ± standard error of the mean [SEM]).

First, we infected Jurkat T cells with HIV_GKO_, a dual reporter HIV-1 virus. A schematic overview of our experiment is shown in Fig. 1A. Latently infected cells are labeled as mKO2 single positive and productively infected cells are labeled as mKO2 and csGFP double positive (Fig. 1B). Four days after infection, we used fluorescence-activated cell sorting (FACS) to sort the cells into three fractions: non-infected (double negative), latently infected (mKO2 single positive), and productively infected (csGFP and mKO2 double positive) cells and confirmed that the purity of each cell fraction was more than 99%.

Next, to confirm that the mKO2-single-positive cells are truly latently infected cells, we measured three HIV transcripts (unspliced [US], single spliced [SS], and multiple spliced [MS]) by reverse transcription-quantitative PCR (RT-qPCR) (6) and the relative copy number of integrated viral DNA by Alu-PCR-based quantitative PCR in each fraction. As expected, productively infected cells showed high levels of all HIV transcripts, while non-infected and latently infected cells showed very low levels (Fig. 1C). In addition, the relative copy numbers of integrated HIV_GKO_ in latently and productively infected cells were comparable (Fig. 1D). These results confirmed that HIV_GKO_ was integrated into the genome but transcriptionally silent in mKO2-single-positive latently infected cells. We also detected HIV-1 integration in 1.3% of double-negative cells using Alu-based quantitative genomic PCR (Fig. 1D), suggesting that a small number of our non-infected cells contain HIV_GKO_ proviruses in which both parts of the HIV-1 LTR and EF1α promoters are deleted. Therefore, we designated these non-infected cells, as a majority of cells (more than 98%) were non-infected and very few cells contained defective HIV DNA.

We next evaluated the reactivation capacity of latently infected cells using three latency-reversing agents (LRAs): tumor necrosis factor alpha (TNF-α), phorbol myristate acetate (PMA), and vorinostat. Latently infected cells were treated with these LRAs for 24 h, and the efficiency of reactivation was assessed by flow cytometry. Whereas around 20% of cells spontaneously reactivated in dimethyl sulfoxide (DMSO)-treated cells, all three LRAs efficiently reactivated latently infected cells (Fig. 1E). This result confirmed that these latently infected cells contain inducible proviruses.

### CAGE captures the transcription start site of HIV_GKO_ at single-base resolution.

**FIG 2:**
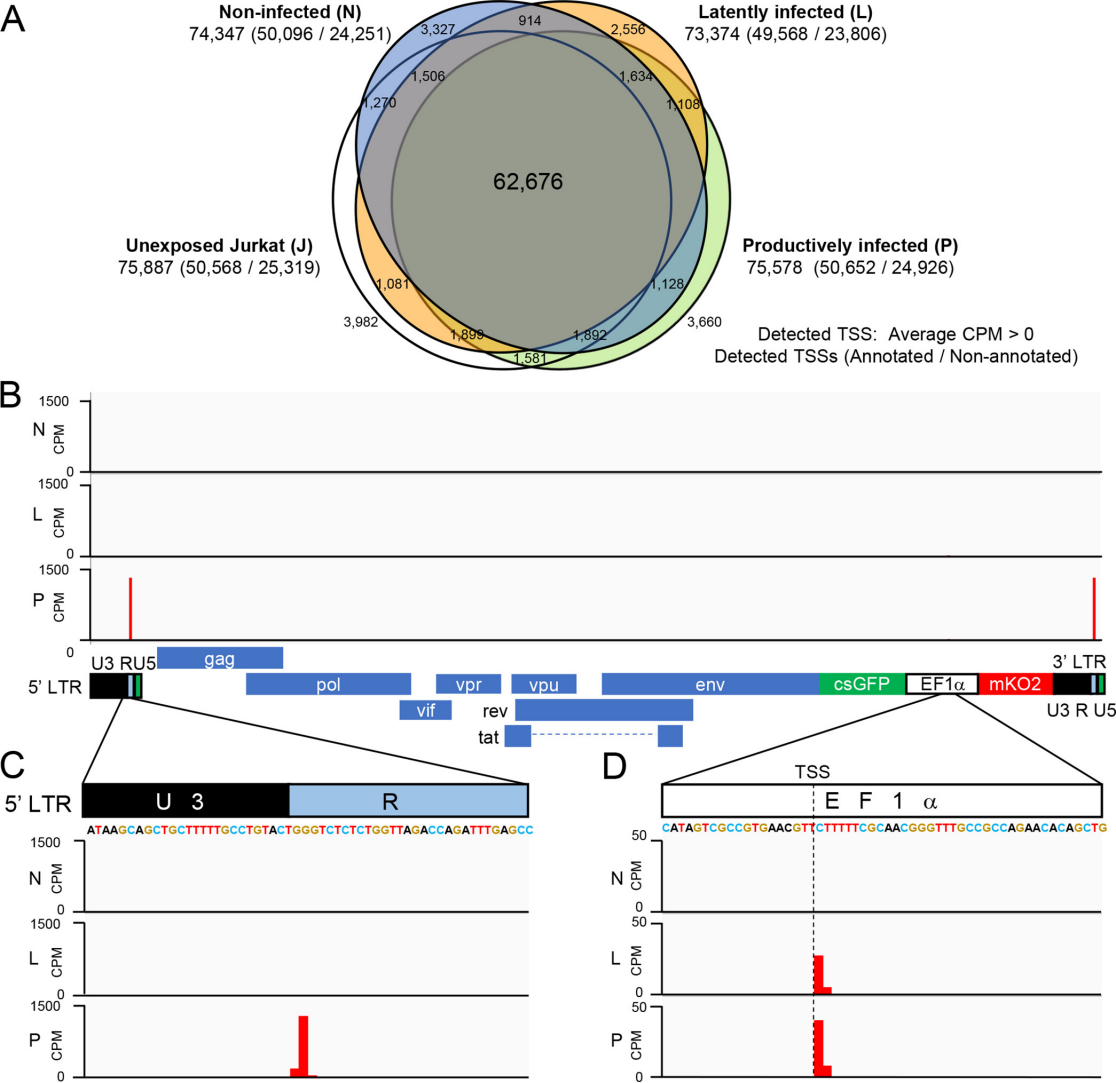
Overview of all detected CAGE tags and HIV derived CAGE tags. (A) Venn diagram of the number of TSSs detected by CAGE in each cell fraction (*n* = 2 biologically independent samples). Detected TSSs were defined as TSSs with an average counts per million (CPM) greater than zero. TSSs that are annotated to specific gene promoters in the FANTOM5 database are defined as annotated, and the remaining TSSs are defined as nonannotated. (B) Integrative Genomics Viewer (IGV) display of normalized mapped read counts (CPM) along the HIV_GKO_ genome for each fraction (N, non-infected; L, latently infected; P, productively infected cells). The genome structure of HIV_GKO_ is shown at the bottom. (C) Enlarged IGV view of the U3/R boundary in the 5′ LTR and showing the normalized mapped read counts in this region for each fraction. (D) Enlarged IGV view of the EF1α locus showing the normalized mapped read counts in this region for each fraction.

We performed CAGE for each FACS-sorted HIV_GKO_ non-infected, latently infected, productively infected, and HIV_GKO_ unexposed Jurkat T cells. We obtained approximately 75 million CAGE tags from 75,000 transcription start sites (TSSs) for each fraction (Fig. 2A). Among these, we detected 380,000 HIV-1-derived CAGE tags, which constituted 0.49% of all detected CAGE tags. The genomic structures of HIV_GKO_ and detected CAGE counts are shown in Fig. 2B. We identified the highest CAGE peak at the 5′ LTR in productively infected cells and also identified high CAGE peaks at the EF1α locus in both latently infected and productively infected cells (Fig. 2C and D). This result also supports that mKO2-single-positive cells were HIV-1 transcriptionally silent. Notably, the highest CAGE peak in the 5′ LTR was located at the U3/R boundary (Fig. 2B), which is consistent with a previous study (26).

### HIV-1 latent infection does not trigger unique cellular responses.

**FIG 3:**
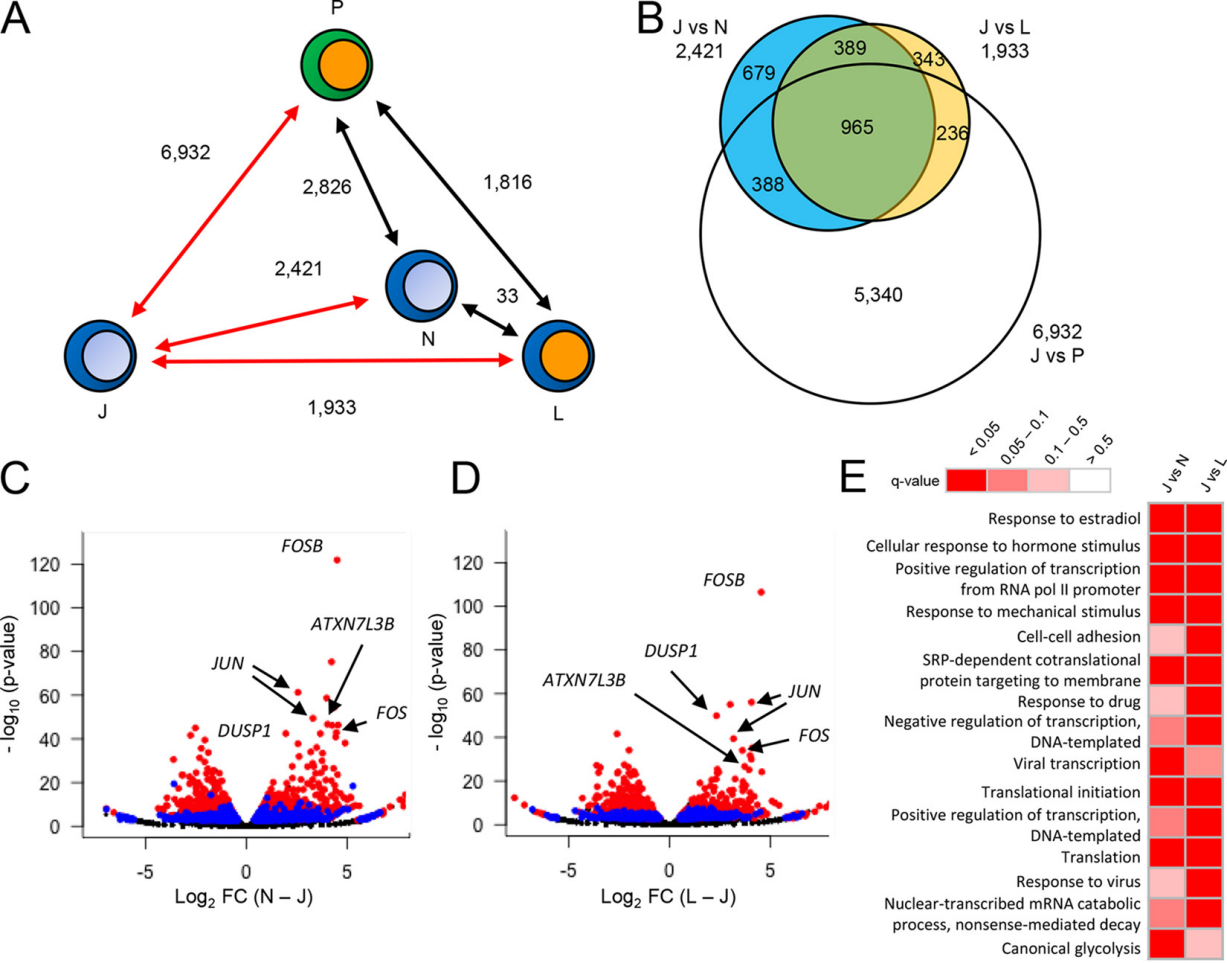
Significantly differentially expressed TSSs between latently infected and HIV_GKO_ nonexposed Jurkat T cells are common in the other comparisons. (A) Numbers of the significantly differentially expressed TSSs when comparing the cell fractions. (B) Venn diagram of the number of significantly differentially expressed TSSs in each comparison. Volcano plots of differentially expressed TSSs between unexposed Jurkat cells and non-infected cells (C) or latently infected cells (D) with log *P* value and log fold change graphed. Significantly differentially expressed TSSs that are common in all three comparisons (J versus N, J versus L, and J versus P) are shown in red; 679 and 343 TSSs that are specifically significant in the comparison of J versus N and J versus L, respectively, are shown in blue. (E) Heat map of gene ontology (GO) enrichment analysis of significantly differentially expressed genes in each comparison. Significantly enriched GO terms (false-discovery rate [FDR] < 0.05) in each comparison are shown.

We next compared CAGE tags from each of the sorted samples with those of unexposed Jurkat cells and found that 965 TSSs were common among all three comparisons (Fig. 3A and B). Furthermore, most of the highly significant TSSs in the comparison between unexposed and latently infected Jurkat cells were common in all three comparisons (Fig. 3C and D). These results suggest that the differences between unexposed Jurkat cells and latently infected Jurkat cells were not unique but rather common in non-infected cells and productively infected cells. DAVID GO analysis (27, 28) showed that significantly enriched biological processes between unexposed and latently infected cells were very similar to those between unexposed and non-infected cells (Fig. 3E), suggesting that the infection and consecutive responses of productively infected cells may have led to stimulation and consecutive similar gene expression changes in both non-infected and latently infected cells while the cells were cultured together. Therefore, we decided to focus on the differences between non-infected, latently infected, and productively infected cells.

**FIG 4:**
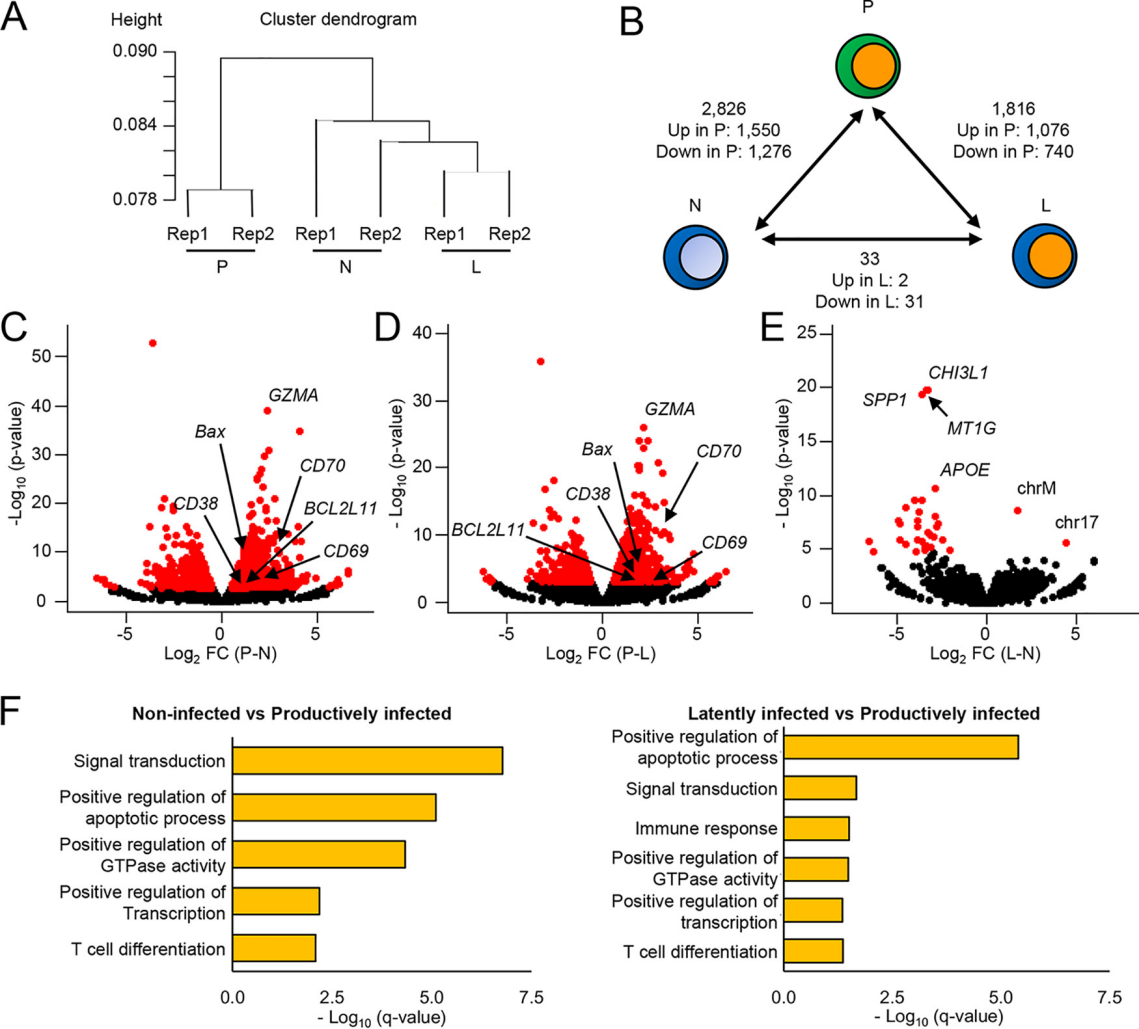
Differential TSS expression in latently infected cells. (A) Cluster dendrogram of CAGE results for non-infected (N), latently infected (L), and productively infected (P) cells, using Spearman’s rank order correlation (*n* = 2 biologically independent samples). (B) Numbers of significantly differentially expressed TSSs (*q *< 0.05) when comparing cell fractions. Volcano plots of differentially expressed TSSs between non-infected and productively infected cells (C) or latently infected and productively infected cells (D), with log *P* value and log fold change graphed. Significantly differentially expressed TSSs are shown in red. Arrows indicate genes associated with T-cell activation and apoptosis. (E) Volcano plot of differentially expressed TSSs between non-infected and latently infected cells with log *q* value and log fold change graphed. Significantly differentially expressed TSSs are shown in red. The top four significantly upregulated genes in non-infected cells and the top two significantly upregulated TSSs in latently infected cells are indicated. (F) Enrichment analysis of the upregulated genes in virus-producing cells compared to those in non-infected or latently infected cells. Representative GO terms in each comparison are shown.

**FIG 5:**
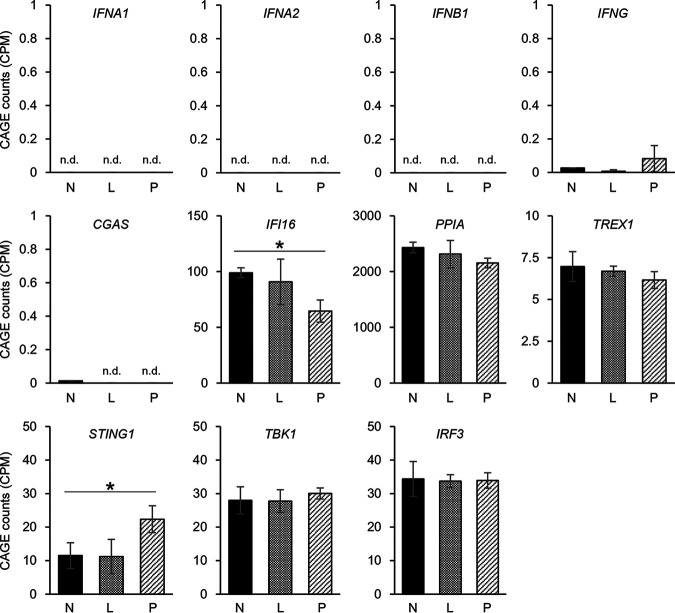
Expression levels of interferon-related genes by CAGE. Normalized expression levels of genes involved in IFN response are shown. n.d., not detected in CAGE; *, *q *< 0.05.

To investigate the differences in gene expression profiles between these three fractions, we performed hierarchical clustering. The gene expression profile of productively infected cells was quite different from that of the other two fractions (Fig. 4A). Notably, we found no major differences between non-infected and latently infected cells. Next, we performed differential expression analysis to identify significant TSSs among non-infected, latently infected, and productively infected cells. Differential expression analysis identified 2,826 TSSs in the comparison between non-infected and productively infected cells and 1,816 TSSs in the comparison between latently infected and productively infected cells, but only 33 TSSs were identified in the comparison between non-infected and latently infected cells (Fig. 4B and see Tables S1, S2, and S3 in the supplemental material). The number of significantly differentially expressed TSSs between non-infected and latently infected cells was much smaller than that between productively infected and non-infected or latently infected cells. We further investigated the expression levels of individual genes that are associated with IFN response. We did not observe any significant upregulation of IFN genes (encoding IFN-α, IFN-β, and IFN-γ) and genes that are associated with IFN response (*CGAS*, *PPIA*, *TREX1*, *IRF3*, and *TBK1*) in latently infected cells or productively infected cells compared to that in non-infected cells; however, we detected a significant decrease of *IFI16* and a significant increase of *STING* expression in productively infected cells (Fig. 5). Altogether, these results suggest that HIV-1 productive infection affects genes involved in the pattern recognition receptor (PRR) signaling pathway; however, HIV-1 latent infection does not trigger any unique cellular transcriptional changes, including innate immune responses.

### Productive infection induces genes associated with apoptosis and T-cell activation.

Among the significant TSSs identified by differential expression analysis, 1,550 TSSs and 1,076 TSSs were upregulated and 1,276 TSSs and 740 TSSs were downregulated in productively infected cells compared to non-infected and latently infected cells, respectively (Fig. 4B and Tables S1 and S2). We found that the expression levels of T-cell activation markers (*CD69*, *CD70*, and *CD38*) were significantly increased in productively infected cells (Fig. 4C and D). Furthermore, we observed an induction of proapoptotic genes (*GZMA*, *BAX*, and *BCL2L11*) in productively infected cells (Fig. 4C and D). Next, to comprehensively investigate which biological processes these upregulated TSSs in productively infected cells belong to, we performed DAVID GO analysis. We confirmed that the upregulated TSSs in productively infected cells belong to T-cell signal transduction, immune response, and apoptosis pathways (Fig. 4F). These results suggest that T-cell activation is required for efficient virus production and that productive infection induces proapoptotic genes, resulting in cell death.

### APOE and SPP1 restrict HIV_GKO_ infection in both Jurkat T cells and primary CD4^+^ T cells.

**FIG 6:**
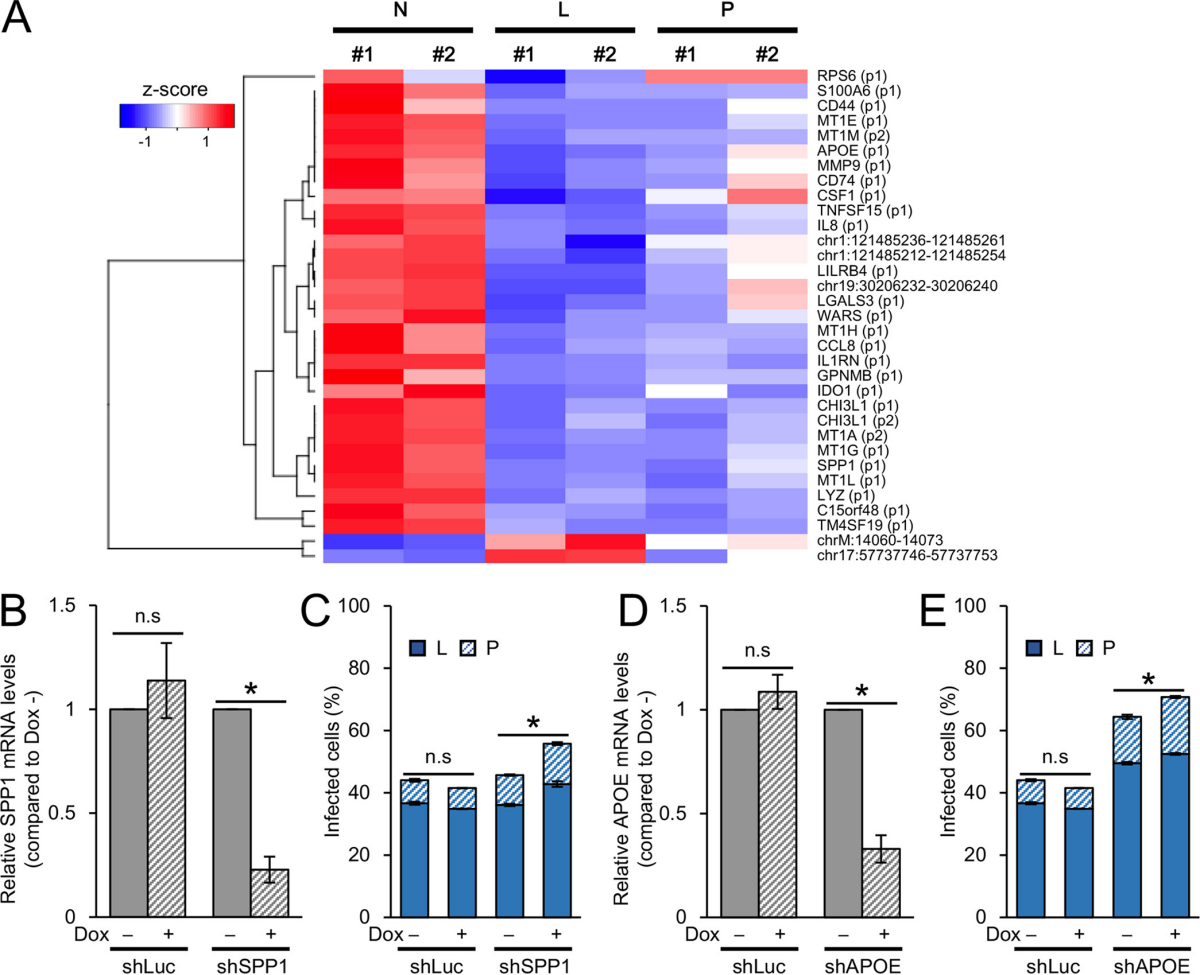
Differentially expressed genes have an antiviral property. (A) Heat map of the 33 significantly (*q *< 0.05) differentially expressed TSSs between non-infected and latently infected cells. (B) RT-qPCR of SPP1 in Jurkat T cells stably transduced with shRNA against luciferase (nontarget control) or SPP1 and treated with or without doxycycline for 4 days. Results are presented relative to expression in doxycycline-untreated controls (*n* = 2, mean ± SD). *, *P* < 0.05, Student’s *t* test. (C) Percentage of latently infected (L) and productively infected (P) cells after SPP1 knockdown (*n* = 2, mean ± SEM). Jurkat T cells stably transduced with shRNA against luciferase or SPP1 were treated with or without doxycycline for 4 days and infected with HIV_GKO_; infection rates were analyzed 2 days postinfection. *, *P* < 0.05, Student’s *t* test. (D) RT-qPCR of APOE in Jurkat T cells stably transduced with shRNA against luciferase (nontarget control) or APOE and treated with or without doxycycline for 4 days. Results are presented relative to expressions in doxycycline-untreated controls (*n* = 2, mean ± SD). *, *P* < 0.05, Student’s *t* test. (E) Percentages of latently infected (L) and productively infected (P) cells after APOE knockdown (*n* = 2, mean ± SEM). Jurkat T cells stably transduced with shRNA against luciferase or APOE were treated with or without doxycycline for 4 days and infected with HIV_GKO_; infection rates were analyzed 2 days postinfection. *, *P* < 0.05, Student’s *t* test.

Differential expression analysis identified 33 significant TSSs between non-infected and latently infected cells. Among these 33 TSSs, only 2 TSSs were upregulated in latently infected cells, but these TSSs were located within nonannotated regions and did not express functional proteins (Fig. 4E and 6A and Table S3). Therefore, we could not identify any specific biomarker for latently infected cells. On the other hand, as illustrated in Fig. 6A, the expression levels of the other 31 TSSs were higher in non-infected cells than in both latently and productively infected cells. We assumed that these upregulated genes in non-infected cells might have putative antiretroviral activity for HIV-1. Among them, we focused on *APOE* and *SPP1*, which both showed significant enrichment in non-infected cells. Apolipoprotein E (APOE) is involved in lipoprotein metabolism and is reported to inhibit HIV-1 infection in macrophages (29, 30). SPP1, also known as osteopontin, a component of the extracellular matrix of bone, suppresses T-cell activation (31, 32) and promotes HIV-1 infection in macrophages (33). To test if these proteins have antiviral properties, we knocked down SPP1 and APOE in Jurkat T cells and evaluated the changes in proportion of HIV_GKO_-infected cells by flow cytometry. First, we established Jurkat T cell lines that express short hairpin RNA (shRNA) against SPP1, APOE, or luciferase (nontarget control) in a doxycycline (DOX)-inducible manner. We confirmed that DOX treatment for 4 days knocked down SPP1 and APOE (Fig. 6B and D). Next, we infected these cells lines with HIV_GKO_, with or without prior DOX treatment, and analyzed the proportion of infected cells by flow cytometry at 2 days postinfection. We found that knockdown of SPP1 and APOE significantly increased the percentage of infected cells (Fig. 6C and E).

**FIG 7:**
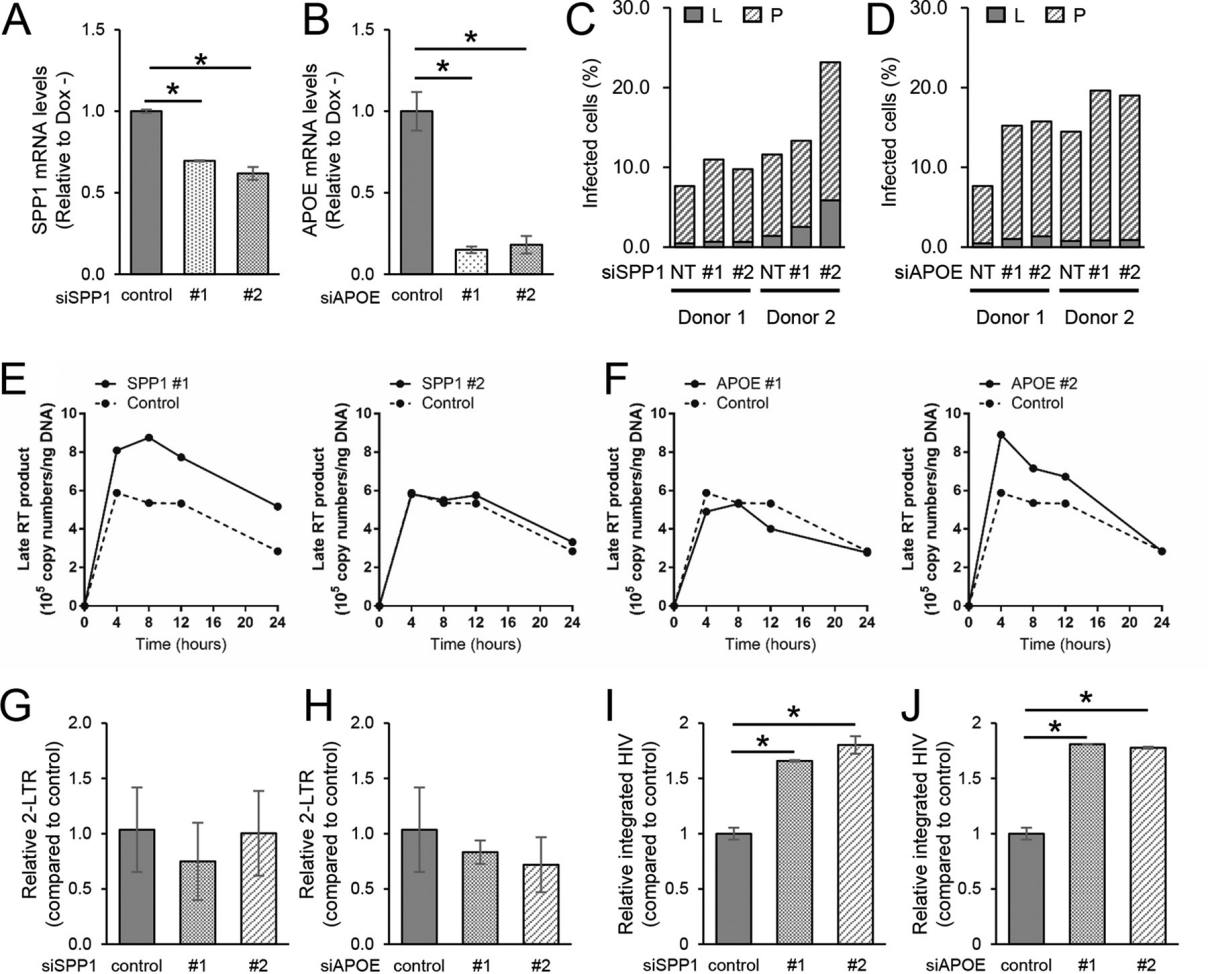
Depletion of SPP1 or of APOE promotes viral replication in primary CD4^+^ T cells. (A) RT-qPCR of SPP1 in primary CD4^+^ T cells transfected with nontarget control siRNA or siRNA against SPP1 number 1 or number 2. Results are presented relative to expression levels in nontarget controls (*n* = 2, mean ± SD) *, *P* < 0.05, Student’s *t* test. (B) RT-qPCR of APOE in primary CD4^+^ T cells transfected with nontarget control siRNA or siRNA against APOE number 1 or number 2. Results are presented relative to expression levels in nontarget controls (*n* = 2, mean ± SD). *, *P* < 0.05, Student’s *t* test. (C) Percentages of latently infected (L) and productively infected (P) cells after SPP1 knockdown. Primary CD4^+^ T cells were stimulated with anti-CD3/28 beads and then transduced with siRNA and infected with HIV_GKO_. Infection rates were analyzed at 2 days postinfection. Results from 2 donors are shown. (D) Percentages of infected cells (latently and productively) after APOE knockdown. Primary CD4^+^ T cells were stimulated with anti-CD3/28 beads and then transduced with siRNA and infected with HIV_GKO_. Infection rates were analyzed at 2 days postinfection. Results from 2 donors are shown. Time course of late RT products when SPP1 (E) or APOE (F) was knocked down in primary CD4^+^ T cells. Primary CD4^+^ T cells were stimulated with anti-CD3/28 beads and then transduced with siRNA against control or target genes and infected with HIV_GKO_. Late RT products at each time point (0, 4, 8, 12, and 24 h) were quantified by qPCR. Results of donor 1 are presented as copy numbers per 1 ng DNA (*n* = 2, mean ± SD). Quantitative PCR results of 2-LTR circles upon SPP1 (G) and APOE (H) knockdown. 2-LTR circles at 24 h postinfection were quantified by qPCR. Results are presented as relative copy number to control (*n* = 2, mean ± SD). Quantitative PCR results of integrated HIV upon SPP1 (I) and APOE (J) knockdown. Integrated HIV at 24 h postinfection was quantified by qPCR. Results are presented as copy number relative to control (*n* = 2, mean ± SD) *, *P* < 0.05, Student’s *t* test.

To further confirm these findings in primary CD4^+^ T cells, we used small interfering RNA (siRNA) to knock down SPP1 and APOE in human primary CD4^+^ T cells and examined infectivity of HIV_GKO_ by flow cytometry. We isolated primary CD4^+^ T cells from peripheral blood and stimulated them with anti-CD3/CD28 beads for 3 days. We introduced siRNA against SPP1 and APOE into stimulated CD4^+^ T cells via electroporation and infected them with HIV_GKO_ at 3 days posttransfection. We evaluated the proportion of HIV_GKO_-infected cells by flow cytometry at 3 days postinfection. We confirmed the knockdown of SPP1 and APOE with RT-qPCR (Fig. 7A and B). As expected, knockdown of SPP1 and APOE increased the proportion of infected cells in human primary CD4^+^ T cells (Fig. 7C and D). Taken together, these results support our hypothesis that SPP1 and APOE have an antiretroviral effect against HIV-1.

To investigate how SPP1 and APOE inhibit HIV infection, we evaluated the amounts of late reverse transcription (RT) products, 2-LTR circles, and integrated HIV upon knockdown of APOE and SPP1 in primary CD4^+^ T cells. Briefly, we introduced siRNA and infected cells with HIV_GKO_ as described above and measured the copy numbers of late RT and 2-LTR products at 4, 8, 12, and 24 h postinfection and of integrated HIV at 24 h postinfection by Alu-qPCR. We detected an increase of late RT products (Fig. 7E and F), relatively low levels of 2-LTR circles (Fig. 7G and H), and a significant increase of integrated HIV upon either SPP1 or APOE knockdown (Fig. 7I and J). These results suggest that both SPP1 and APOE prevent HIV infection by interfering with integration and possibly RT.

### Increased phosphorylation of S6K helps prevent latency.

**FIG 8:**
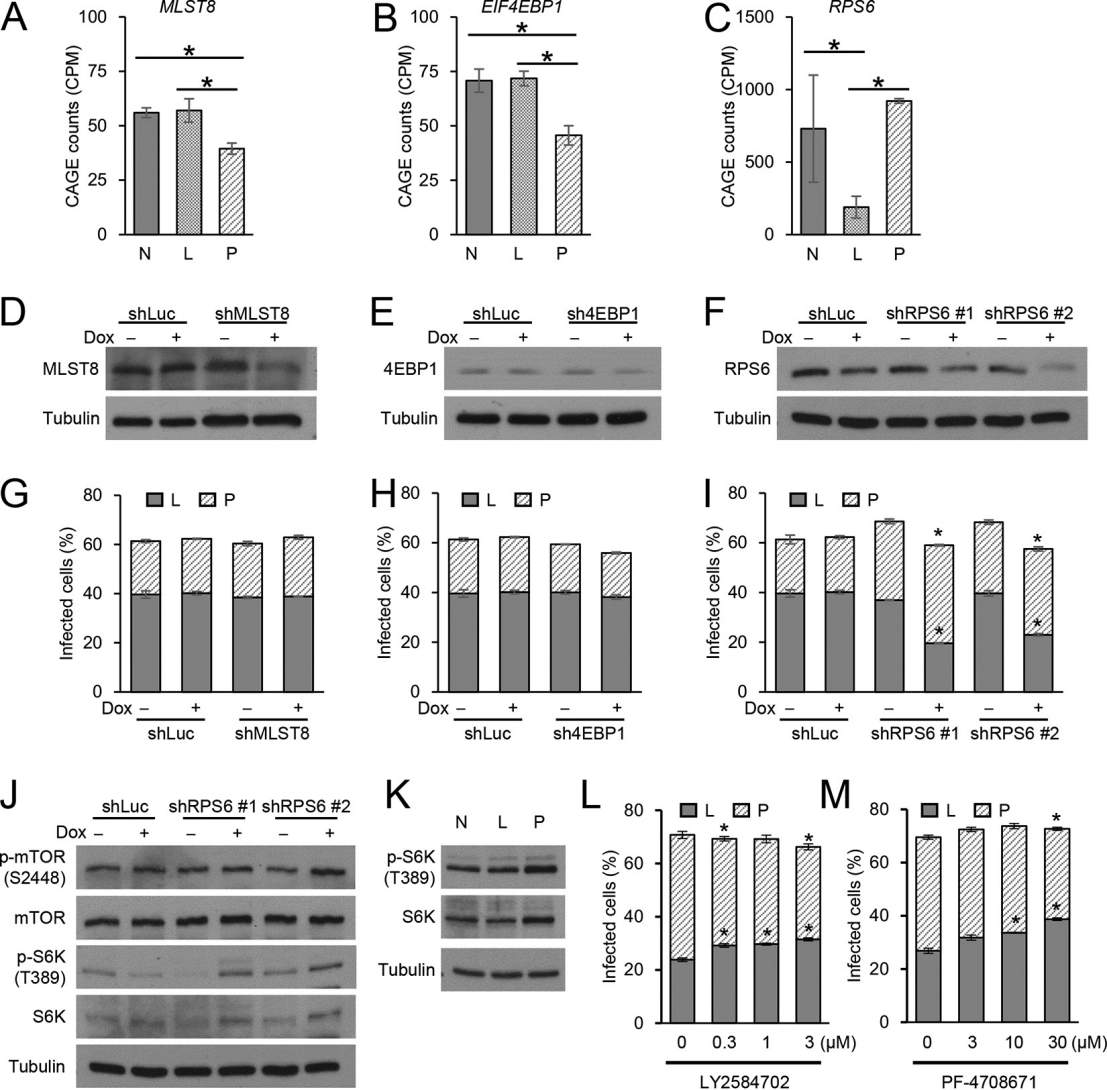
Impact of the mTOR signaling pathway in establishing the latent reservoir. (A to C) CAGE results at single-gene level. The normalized expression levels of three significantly differentially expressed genes (*, *q *< 0.05) that belong to the mTOR signaling pathway are shown. Target gene immunoblotting results of Jurkat T cells stably transduced with shRNA against luciferase (nontarget control), MLST8 (D), EIF4EBP1 (E), or RPS6 (F) treated with or without doxycycline for 4 days. (G to I) Percentages of latently infected (L) and productively infected (P) cells after knockdown of each gene shown in panels A to C (*n* = 2, mean ± SEM). Jurkat T cells stably transduced with shRNA against luciferase (nontarget control), MLST8 (G), EIF4EBP1 (H), or RPS6 (I) were treated with or without doxycycline for 4 days and infected with HIV_GKO_; infection rates were analyzed 4 days postinfection. *, *P* < 0.05, Student’s *t* test. (J) mTOR, S6K, and phosphorylated mTOR (p-mTOR, S2448) and S6K (p-S6K, T389) immunoblotting results of cells treated as for panels G to I. (K) p-S6K (T389) and S6K immunoblotting results of sorted HIV_GKO_-infected Jurkat T cells. Tubulin was used as a loading control in images in panels D to F, J, and K. (L and M) Jurkat T cells were infected with HIV_GKO_, incubated for 24 h, and then treated with specific S6K inhibitors (LY2584702 [L] and PF-4708671 [M]), and the percentages of latently and productively infected cells were assessed at 4 days postinfection. *, *P* < 0.05, Student’s *t* test.

**FIG 9:**
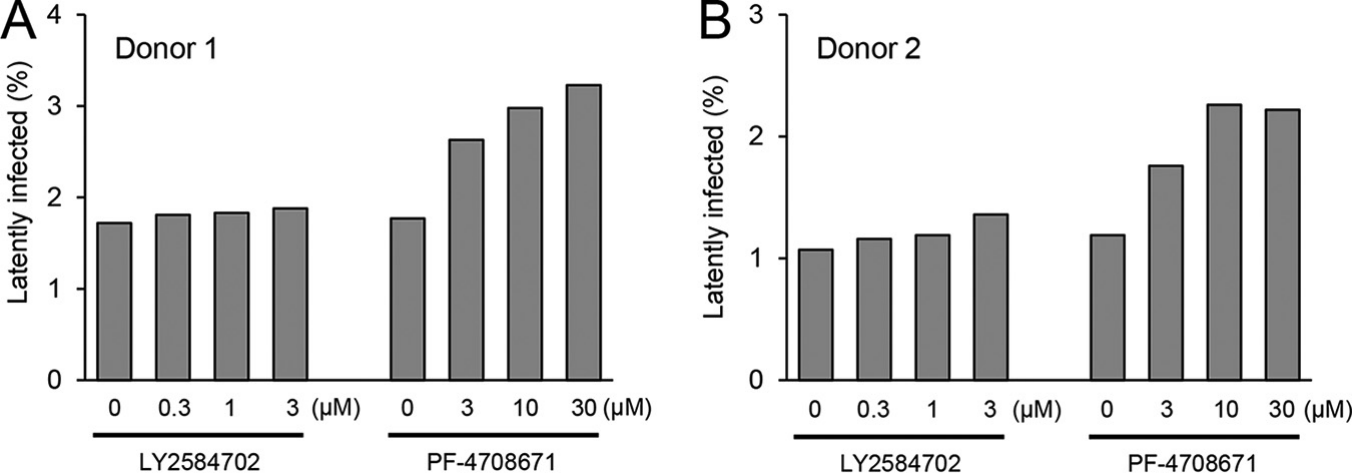
S6K inhibition promotes establishment of latency in primary CD4^+^ T cells. Primary CD4^+^ T cells were stimulated with anti-CD3/28 beads for 3 days and infected with HIV_GKO_. At 24 h postinfection, CD4^+^ T cells were treated with specific S6K inhibitors (LY2584702 or PF-470861), and the percentages of latently infected cells were evaluated at 4 days postinfection. Results from donor 1 (A) and donor 2 (B) are shown.

The mammalian target of rapamycin (mTOR) signaling pathway is associated with the regulation of cell proliferation as well as lipid and amino acid metabolism (34). Genome-wide functional screening using an shRNA library revealed that the suppression of the mTOR complex prevents CD3/CD28-mediated reactivation of latently infected cells and mTOR inhibitors suppress CD3/CD28-mediated reactivation of latently infected cells (35). In addition, genetic depletion of TSC1, which inhibits the activation of the mTORC1 complex through the phosphorylation of Rheb, promotes the reactivation of latently infected cells (36). These results suggest that the mTOR signaling pathway plays a role in reactivating latently infected cells, but it remains unknown whether mTOR also plays a role in establishing the latent reservoir. We investigated the expression levels of genes that compose the mTOR signaling pathway and found that there were significant differences in the expression levels of *MLST8*, *EIF4EBP1*, and *RPS6* between latently infected and productively infected cells (Fig. 8A, B, and C, respectively), suggesting that the activity of the mTOR signaling pathway has some influence on establishing latency. To confirm this possibility, we established cell lines that stably express shRNA against MLST8, 4EBP1, or RPS6 in a doxycycline-inducible manner and confirmed efficient knockdown of each protein after 4 days of DOX treatment (Fig. 8D, E, and F, respectively). We then infected these cells with HIV_GKO_ and evaluated the proportion of infected cells by flow cytometry. We observed that knockdown of RPS6 decreased the percentage of latently infected cells and increased that of productively infected cells, while the overall infection (latent plus productive infections) rate was largely the same. However, the knockdown of MLST8 and 4EBP1 had no effect on infection rates or type (Fig. 8G, H, and I). Based on these results, we hypothesized that knockdown of RPS6 would increase the phosphorylation of upstream components of the mTOR signaling pathway and examined the phosphorylation state of mTOR and S6K. As expected, knockdown of RPS6 increased the expression and phosphorylation of S6K (Fig. 8J). We observed no differences in the expression or phosphorylation of mTOR upon knockdown of RPS6 (Fig. 8J). Furthermore, we found that productively infected cells had higher levels of phosphorylated S6K than non-infected and latently infected cells (Fig. 8K). Expression of S6K itself was also increased, possibly because phosphorylation of S6K at T389 stabilizes S6K itself by preventing proteasome degradation induced by JNK1-mediated phosphorylation of S6K at S424 (37). S6K phosphorylates CDK9, a component of the p-TEFb complex, and plays an important role in the reactivation of latently infected cells (35). We used selective S6K inhibitors (LY2584702 and PF-4708671) to investigate whether S6K has an impact on establishing the latent reservoir. Both S6K inhibitors increased the size of the latent reservoir (Fig. 8L and M). The increase of the HIV latently infected cells by S6K inhibition was also confirmed in primary CD4^+^ T cells (Fig. 9). Taken together, these results suggest that HIV infection tends to become productive when the phosphorylation of S6K in infected cells is relatively high, while it tends to become latent when it is at a relatively low level.

## DISCUSSION

In this study, we demonstrate that latent HIV infection does not trigger a unique cellular response in T cells, while, on the other hand, productive infection enhances T-cell activation and induces proapoptotic responses. In addition, we show that among the genes that are upregulated in non-infected cells compared to that in latently infected cells, APOE and SPP1 have antiretroviral properties in T cells. Finally, we reveal that phosphorylation of S6K is increased in productively infected cells and that inhibiting S6K suppresses productive infection and promotes latent infection.

A prior study using HIV-infected cells derived from untreated viremic patients showed that infected cells express ICOS, a critical costimulator of follicular helper T cells, indicating recent cell activation (38). Bradley et al. also reported T-cell activation genes were upregulated in productively infected cells (21). In line with these findings, we observed the upregulation of some T-cell activation markers in productively infected cells. In addition, it is widely accepted that productive infection results in apoptotic cell death through a viral cytopathic effect and is thought to be a mechanism that kills reactivated cells (39–41). Consistent with these reports, we found that HIV infection causes upregulation of proapoptotic genes in productively infected cells but not in latently infected cells. A recent publication demonstrated that the antiapoptotic protein BIRC5 and its upstream regulator OX40 were upregulated in both productively and latently infected CD4^+^ T cells and can promote survival of HIV-1-infected CD4^+^ T cells (42). In contrast, we observed the downregulation of OX40 in productively infected cells (data not shown), but its expression was maintained in latently infected cells. Overall, our results together with these various observations suggest that productive infection leads to an apoptotic response in infected cells, but latent infection allows cells to escape from such a response and survive to form the latent reservoir.

Two pattern recognition receptors (PRRs), IFI16 and cGAS, play central roles in sensing HIV-1 and inducing subsequent interferon responses (10, 11). These PRRs recognize HIV-1 reverse transcription (RT) products and then activate STING and induce interferon responses (10, 11). On the other hand, HIV-1 capsid prevents host innate immune responses by interacting with CypA and CPSF6, allowing RT products to escape from being recognized by these PRRs (12–14). The HIV-1 accessory protein Vpu also suppresses host innate immune responses by degrading interferon regulatory factor 3 (IRF3), a central transcription factor that drives host cell innate immunity (43). Moreover, cytosolic exonuclease TREX1 degrades excess RT products and supports HIV-1 to evade innate immune recognition and successfully infect the host cell (44). Therefore, it remains unknown whether HIV latent infection triggers innate immune responses. In this study, we did not observe innate immune responses in latently infected or productively infected cells. This could be in part because we used a vesicular stomatitis virus G (VSV-G) pseudotyped replication-incompetent single-round infection system, while Vermeire et al. used a spreading infection system to report that HIV-1 infection induces interferon responses in primary CD4^+^ T cells (45). As a result, we may have underestimated the innate immune response against HIV-1. Overall, these results provide new insights into the host innate immune response against latent HIV infection.

SPP1 is known as a chemoattractant that promotes the migration of macrophages, and it is also known as a proinflammatory cytokine that enhances Th1 cytokine production (46). The plasma and cerebrospinal fluid levels of SPP1 are well correlated with the neuropsychological status of HIV-1-infected patients (47). We found that *SPP1* was upregulated in non-infected cells, suggesting that the presence of SPP1 induces distant antiviral responses that protect cells from further infection. We also found that knockdown of SPP1 increased the susceptibility of T cells to HIV-1 infection. In contrast, a previous study showed that SPP1 promotes HIV-1 infection in human-derived macrophages through the downregulation of IκBα and subsequent activation of NF-κB (33). Our results suggest that SPP1 prevents HIV infection by interrupting RT or integration and plays a different role in T cells than in macrophages. In addition, SPP1 is a cytokine and ligand for integrin αvβ5 and CD44, and further loss of SPP1 increases the activity of CD4 and CD8 T cells (31, 32). Therefore, SPP1 may indirectly prevent HIV-1 infection through these cytokine signals. Altogether, our findings shed light on the role of SPP1 in HIV infection, but further investigation is required to fully elucidate the role of SPP1 in HIV-1 infection.

Apolipoprotein E (APOE) is a small secreted protein involved in cellular lipid metabolism and cholesterol transport. The relationship between *APOE* polymorphisms and the development of Alzheimer’s disease is well studied, and the *APOE* E4 allele is a risk factor for Alzheimer’s disease. The relationship between *APOE* polymorphisms and HIV-associated neurocognitive disease (HAND) was also extensively studied, but the results are still controversial (48–53). Regarding the relationship between APOE and HIV infectivity, one report showed that the APOE E4 isoform protein enhances HIV-1 infection (54). In general, however, APOE is known as a restriction factor of HIV, and various studies have proposed various mechanisms of how APOE inhibits HIV infection (29, 30, 55, 56). Among these, two studies showed that APOE disrupted HIV infection at the HIV entry or RT steps. In the present study, we found that the expression of APOE was increased in non-infected cells compared to that in latently infected and productively infected cells, and knockdown of APOE promoted HIV-1 infection in T cells by interfering with viral RT or integration. Thus, our findings are consistent with previous studies that support the notion that APOE is an HIV-1 restriction factor.

The mTOR signaling pathway plays a central role in coordinating cell growth and metabolism with extracellular stimuli (34, 57). mTOR inhibitors perturb HIV-1 infection by inhibiting various steps of the HIV-1 life cycle (58, 59). Thus, the mTOR pathway plays a role in efficient HIV-1 infection. Recently, Besnard et al. identified the mTOR complex as a modulator of HIV latency by conducting genome-wide screening with a pooled ultracomplex shRNA library and revealed that mTOR inhibitors suppressed reactivation of latently infected CD4^+^ T cells (35). Moreover, several studies showed that suppression of the mTOR signaling pathway prevents reactivation of latently infected cells (36, 60). Altogether, it seems that the mTOR signaling pathway supports efficient virus production, and inhibition of mTOR promotes HIV latency. However, it remains unclear whether low activity of the mTOR signaling pathway influences latency establishment. We demonstrated that knockdown of RPS6 upregulated the mTOR signaling pathway through increased phosphorylation of S6K and decreased the proportion of latently infected cells. We further identified that S6K phosphorylation increased in productively infected cells and that S6K inhibitors suppress productive infection and promote latent infection. S6K is known to phosphorylate CDK9, which is a component of the p-TEFb complex that serves as a cofactor of Tat-mediated transcription (35, 61). These findings suggest that the activity of the mTOR signaling pathway, especially the phosphorylation level of S6K, in infected cells has a significant influence not only on reversing latency but also on establishing latency, and suppression of S6K promotes infected cells to become latent. Taken together, these data provide a new mechanistic insight into how the HIV-1 reservoir is established.

In conclusion, we used an *in vitro* latency model that can directly and prospectively differentiate latently infected cells from non-infected and productively infected cells to perform a transcriptome analysis of each of these cellular fractions. We used the CAGE method to perform this analysis, which can precisely analyze the transcriptome at individual transcription start site levels and demonstrated that HIV-1 productive infection enhances T-cell activation and induces proapoptotic responses, while the integrated HIV itself is no longer recognized by host cellular immune systems when the infection becomes latent. We found that *APOE* and *SPP1* are upregulated in non-infected cells and have antiviral properties in T-cells. In addition, we also revealed that the increased phosphorylation of S6K promotes productive infection and thereby inhibition of S6K promotes latent infection. Overall, our study provides a new insight into the cellular response induced by HIV-1 latent infection and the molecular mechanism that is associated with establishing the latent HIV-1 reservoir.

## MATERIALS AND METHODS

### Cell lines and plasmids.

Jurkat T cells were cultured in RPMI medium supplemented with 10% fetal bovine serum (FBS) and 1% penicillin-streptomycin-glutamine (PSG). Primary CD4^+^ T cells were cultured in RPMI medium supplemented with 10% FBS, 1% PSG, and 100 IU/ml human recombinant interleukin 2 (IL-2) (Shionogi & Co.). HIV_GKO_ was described in a previous study (6). Tet-pLKO-puro was purchased from Addgene (plasmid number 21915). We generated Tet-pLKO-puro vectors that express shRNA against luciferase (nontarget control), SPP1, APOE, MLST8, 4EBP1, RPS6 number 1, and RPS6 number 2 by inserting synthesized double-stranded oligonucleotides between the EcoRI and AgeI restriction sites. Sequences of the synthesized oligonucleotides are provided in Table S4 in the supplemental material.

### Virus production.

HIV_GKO_ viral stocks were generated by cotransfecting Lenti-X 293T cells with a plasmid encoding HIV_GKO_ and a plasmid encoding VSV-g envelope. Supernatants were collected 48 h after transfection, centrifuged (2,000 rpm, 20 min, room temperature), filtered through 0.45-μm pore-size polyvinylidene difluoride (PVDF) membranes, and concentrated by ultracentrifugation (25,000 rpm, 2 h, 4°C). Concentrated viral particles were resuspended in RPMI medium and stored at −80°C. Virus concentration was measured using the HIV-1 p24 antigen enzyme-linked immunosorbent assay (ELISA) kit (ZeptoMetrix) according to the manufacturer’s protocol. For the lentivirus expressing shRNAs against luciferase, SPP1, APOE, MLST8, 4EBP1, and RPS6, we cotransfected Lenti-X 293T cells with each Tet-pLKO-puro plasmid described above and the Trans-Lentiviral packaging plasmid mix (GE Dharmacon). Supernatants were collected, concentrated, and stocked as described above.

### HIV_GKO_ infection and sorting.

Jurkat T cells were infected with VSV-g pseudotyped HIV_GKO_ at a concentration of 15 ng of p24 per 3 × 10^5^ cells in 24-well plates. Infected cells were washed twice with phosphate-buffered saline (PBS) at 24 h after infection and analyzed or sorted at 4 or 5 days after infection with a FACS Aria II. Primary CD4^+^ T cells were spinoculated with VSV-g pseudotyped HIV_GKO_ at a concentration of 70 ng of p24 per 1.5 × 10^5^ cells for 2 h at 900 × *g* at 32°C. Infected cells were cultured in the presence of 100 IU/ml IL-2 and analyzed at 3 days postinfection with a FACS Aria II.

### DNA extraction and quantitative PCR.

Genomic DNA was extracted from cells using the QuickGene DNA whole blood kit S (Kurabo) according to the manufacturer’s protocol. Time course determinations of late RT products and quantification of 2-LTR circles were performed as described previously (62). Integrated HIV_GKO_ genome was selectively amplified from the isolated genomic DNA by using a forward primer located in the Alu sequence and a reverse primer located in the Gag sequence of HIV_GKO_. In the 2nd PCR step, the amplified integrated HIV_GKO_ genome was measured by quantitative PCR using primers located in the 5′ LTR of HIV_GKO_. The primer sequences are provided in Table S4.

### RNA extraction and RT-qPCR.

Total RNA was extracted using the High Pure RNA isolation kit (Roche). cDNA was synthesized using ReverTra Ace qPCR RT master mix with genomic DNA (gDNA) remover (Toyobo Life Science) with a combination of random and oligo(dT) primers. Real-Time PCR was performed using TB green premix Ex *Taq* II (TaKaRa). qPCR levels of cell-associated HIV mRNA copy number and expression of APOE and SPP1 were normalized to endogenous glyceraldehyde-3-phosphate dehydrogenase (GAPDH) expression. The primer sequences are provided in Table S4.

### Protein extraction and Western blotting.

Cells were lysed with SDS sample buffer (62.5 mM Tris-HCl, 2% SDS, 2% glycerol). Protein content was determined using the Pierce bicinchoninic acid (BCA) protein assay kit (Thermo Fisher). Whole-cell lysates were subject to immunoblot analysis using anti-MLST8 (GβL) (CST), anti-phospho-4EBP1(T37/46) (CST), anti-4EBP1 (CST), anti-phospho RPS6 (S240/244) (Merck), anti-RPS6 (Abcam), anti-phospho-p70-S6K (T389) (CST), anti-p70-S6K (CST), anti-phospho mTOR (S2448) (CST), anti-mTOR (CST), and anti-tubulin (Sigma) antibodies.

### CAGE library preparation.

Total RNA from each sample was extracted with miRNeasy minikit (Qiagen). CAGE libraries were constructed using total RNAs based on the no-amplification nontagging CAGE libraries for Illumina next-generation sequencers (nAnT-iCAGE) protocol (63). Briefly, first-strand cDNAs were synthesized through reverse transcription using random primers. Subsequently, 5′ caps of RNAs were oxidized and biotinylated. Cells were then treated with RNase I to remove single-stranded RNAs. Biotinylated RNA/cDNA molecules were purified using streptavidin magnetic beads. Single-stranded cDNAs were obtained by treating with RNase H and RNase I. After ligation of adapters and second-strand synthesis of cDNAs, CAGE libraries were finalized. CAGE libraries were sequenced with NextSeq 500 (Illumina) in single-read mode.

### CAGE data analysis.

HIV_GKO_ sequence and human reference genome hg19 were combined, and the index for the combined sequence was generated using HISAT2 version 2.0.5 (64) with the default parameters. Raw reads were trimmed and aligned to the combined sequence using HISAT2 with the following parameters: hisat2 -p 12 –trim3 2 -S. Next, generated SAM files were converted into BAM files, and uniquely mapped reads with high mapping quality values (MAPQ ≥ 20) were retained using SAMtools version 1.9 (65). Using bedtools v2.25.0 (Quinlan, Bioinformatics 2010) with the parameters of genomecov -5 -bg -strand + or genomecov -5 -bg -strand −, coverage of 5′ ends of reads were computed on FANTOM5-defined promoters (25) that were downloaded from http://fantom.gsc.riken.jp/5/datafiles/latest/extra/CAGE_peaks/. The resultant bedGraph files were converted to bigWig files with bedGraphtobigWig (66). Reads mapped to promoters were counted using bigWigAverageOverBed (66). Promoters with no expression in any sample were filtered out. After normalization by relative log expression, differential expression analysis was carried out with edgeR version 3.16.5 (67), and *q* values were calculated using the Benjamini-Hochberg method (68). For alignment to the HIV_GKO_ sequence, each read was allowed to multimap to at most 2 loci, because the 5′ LTR has the same sequence arrangement as the 3′ LTR. Multimapping reads were divided equally among candidates. CAGE signals in the HIV_GKO_ sequence were visualized using Integrative Genomics Viewer (69).

Enrichment analysis was conducted on the identified significantly differentially expressed genes (*P* < 0.05) using DAVID Functional Annotation Tool (27, 28). The resulting *P* values were corrected with the Benjamini-Hochberg method (68).

### Lentiviral shRNA transduction.

To establish Jurkat T cells that were stably transduced with inducible shRNAs against luciferase, APOE, SPP1, MLST8, 4EBP1, RPS6 number 1, and RPS6 number 2, Jurkat T cells were infected with lentivirus containing inducible shRNA against each gene and treated with puromycin 48 h postransduction to select stably transduced cells. The selected cells were maintained in RPMI medium supplemented with 10% FBS, 1% PSG, and 1 μg/ml of puromycin.

### Primary CD4^+^ T cell isolation and activation.

Primary CD4^+^ T cells were isolated from human peripheral blood using RosetteSep human CD4^+^ T cell enrichment cocktail (StemCell Technologies) according to the manufacturer’s protocol. Isolated primary CD4^+^ T cells were stimulated with CD3/CD28 activation beads (Gibco) at a concentration of 0.5 bead/cell in the presence of 100 IU/ml IL-2 for 3 days.

### siRNA electroporation.

siRNAs were purchased from Thermo Fisher Scientific (SPP1, Silencer select predesigned siRNA s13375 and s13376; APOE; Silencer select predesigned siRNA s1495 and s194291; nontarget control; Silencer select negative control number 1 siRNA [4390843]). Purified CD4^+^ T cells were stimulated with CD3/CD28 activation beads for 3 days prior to electroporation. siRNA against each gene or nontarget control was then introduced into activated CD4^+^ T cells via electroporation at a concentration of 300 nM per 1.0 × 10^6^ cells. Electroporation was performed using the T-023 program on the Nucleofector 2b (Lonza). After electroporation, cells were returned to culture and were kept stimulated with CD3/CD28 activation beads until HIV_GKO_ infection.

### Statistical analysis.

Significance in quantitative PCR and flow cytometry was analyzed by Student’s *t* test, and marked with an asterisk in the manuscript.

### Data availability.

Raw and processed data are available from the Gene Expression Omnibus under accession no. GSE149492.
